# Exercise-based pulmonary rehabilitation for a post-COVID-19 pulmonary fibrosis patient: A case report

**DOI:** 10.1097/MD.0000000000027980

**Published:** 2021-11-24

**Authors:** Kyungyeul Choi, Minwoo Kim, Son mi Lee, JongKyu Kim

**Affiliations:** Department of Physical Medicine and Rehabilitation, Seoul Medical Center, Jungnang-gu, Seoul, Republic of Korea.

**Keywords:** coronavirus disease 2019, pulmonary fibrosis, rehabilitation

## Abstract

**Rationale:**

Pulmonary fibrosis is an infamous sequela of coronavirus disease 2019 (COVID-19) pneumonia leading to long-lasting respiratory problems and activity limitations. Pulmonary rehabilitation is beneficial to improve the symptoms of lung fibrosis. We experienced a post-COVID-19 pulmonary fibrosis patient who received a structured exercise-based pulmonary rehabilitation program.

**Patient concerns:**

This article presents a case of successful pulmonary rehabilitation of a patient with post-COVID-19 pulmonary fibrosis. The patient could not cut off the oxygen supplement even after a successful recovery from COVID-19.

**Diagnosis:**

Diagnosis of COVID-19 was based on the reverse transcription-polymerase chain reaction (RT-PCR). Pulmonary fibrosis was diagnosed by patient's complaint, clinical appearance, and computed tomography (CT) on chest.

**Intervention:**

The patient underwent ten sessions of exercise-based rehabilitation program according to Consensus Document on Pulmonary Rehabilitation in Korea, 2015.

**Outcome:**

On the 8th day, he could cut off the oxygen supplementation and complete the one-hour exercise without oxygen. He was discharged after completing the 10-session program without any activity limitations.

**Lessons:**

Exercise-based pulmonary rehabilitation will help the post-COVID-19 pulmonary fibrosis patients. This case suggested the importance of pulmonary rehabilitation program to the post-COVID-19 pulmonary fibrosis patient.

## 1 Introduction

Since the outbreak of the severe acute respiratory syndrome coronavirus 2 (SARS-CoV-2) pandemic, many after effects have been reported from the survivors of coronavirus disease 2019 (COVID-19), termed as long-COVID or post-acute COVID-19 syndrome.^[1]^ Among them, pulmonary fibrosis is an ill-famed sequela that presents with dry cough, fatigue, dyspnea, and the need for oxygen supplementation in severe cases.^[2]^

Pulmonary fibrosis was a complication of severe acute respiratory syndrome (SARS) and middle east respiratory syndrome in previous epidemics. In SARS patients, they first developed atypical pneumonia, followed by acute lung injury and acute respiratory distress syndrome, evolving into fibrosis. Fibrosis occurs more commonly amongst the elderly and in patients with severe and more chronic diseases.^[2]^ High-dose steroids were given routinely to many SARS patients, limiting the incidence of fibrosis. The benefit of dexamethasone in severe COVID-19 patients has been established.^[1,3]^ However, besides pharmacologic treatment, pulmonary rehabilitation might play an important role for post-COVID fibrotic lung diseases. Several studies have suggested the importance of pulmonary rehabilitation.^[4,5]^ However, their rehabilitation protocols are diverse, and a standard rehabilitation approach is not suggested yet.

We provided a standard pulmonary rehabilitation program to a post-COVID lung fibrosis patient who needed oxygen supplementation and reported the outcome.

## 2 Case report

**Figure 1:**
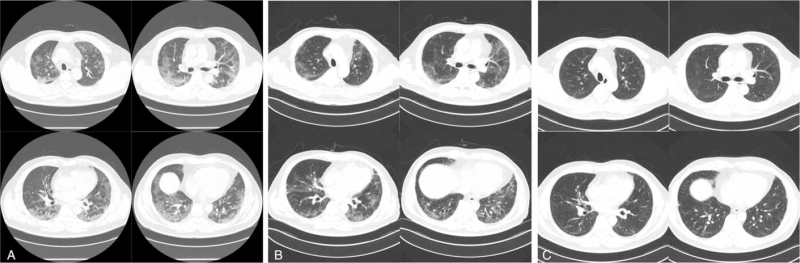
Chest CT images of the case. (A) The first computed tomography (CT) scan at admission day, ground-glass opacities (GGO) were observed in entire lung fields. (B) CT at 47th hospital day showed decreased GGO, but prominent fibrotic lung tissue change was suggested. (C) 3 months follow-up CT after discharge showed a decreased amount of lung fibrosis.

**Figure 2:**
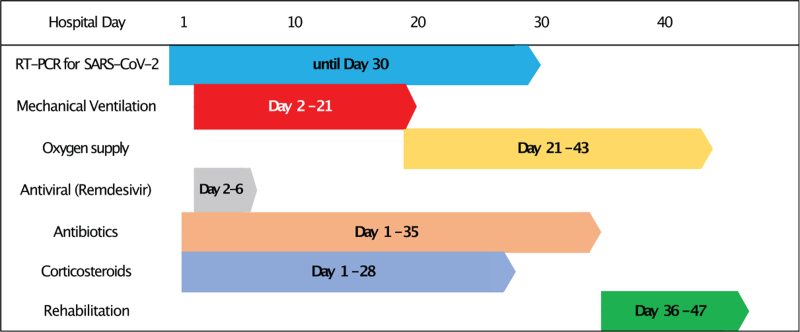
Timeline of COVID-19 treatment. The patient got mechanical ventilation from the second hospital day. After successful weaning off, he required oxygen supplementation. During the 10-session rehabilitation program, he could cut-off the oxygen successfully.

A 59-year-old man was admitted to our hospital with symptomatic COVID-19 for 5 days. The diagnosis was based on a nasopharyngeal swab using the reverse transcription-polymerase chain reaction test for SARS-CoV-2 RNA and chest computed tomography (Fig. 1). Two days after admission, that is, 7 days after symptom onset, his dyspnea worsened, and he required endotracheal intubation. He was subsequently placed on mechanical ventilation for 20 days. On the 30th hospital day, his nasopharyngeal swab PCR test result turned negative, and he was transferred from the intensive care unit to the general ward. The antibiotics and corticosteroids were stopped as the general condition improved. However, he required oxygen supplementation via nasal cannula with a flow of 2 L per minute of oxygen. The pulmonologist diagnosed the patient with pulmonary fibrosis and referred him to the rehabilitation department (Fig. 2).

At the start point of rehabilitation, his motor grades were 5/5 in upper limbs and 4/4 in lower limbs. He could walk independently, but the endurance was limited to 200 m with oxygen supplementation. We decided to provide a conventional pulmonary rehabilitation program for pulmonary fibrosis according to “Consensus Document on Pulmonary Rehabilitation in Korea, 2015”.^[6]^

The program consisted of 10 supervised physical therapy sessions according to Frequency, Intensity, Time, and Type principles: 5 days a week of frequency, 12 to 14 on Borg Rating of Perceived Exertion (RPE) of intensity, 60 minutes per day of time, stretching, strengthening and aerobic exercises of type. To keep the exercise intensity to 12–14 of RPE, the physical therapist checked the RPE and managed the intensity every 10 minutes (see Supplementary Exercise protocol).

At the first session, he could not perform the 1-hour exercise program without oxygen supplementation. However, on the 8th day, he did not require oxygen supplementation during the whole exercise session. The functional evaluation showed improvements (Table 1). Thus, he was discharged without oxygen supplementation on the 49th hospital day.

**Table 1:** Functional improvements of the patient.

	T0	T1	T2
Respiratory functions			
FVC (% predicted)	2.47 L (62%)	2.65 L (65%)	2.8 L (68%)
FEV1 (% predicted)	2.21 L (78%)	2.23 L (78%)	2.36 L (82%)
FEV1/FVC	89	84	84
MIP	65 cm H_2_O	77 cm H_2_O	83 cm H_2_O
MEP	73 cm H_2_O	82 cm H_2_O	115 cm H_2_O
PCF	360 L/min	580 L/min	640 L/min
MMRC	3	2	1
Hand grip strength (right/left)	33/28 (kg)	33/29 (kg)	34/32 (kg)
Functional abilities			
MBI (total 100)	66	91	100
MRMI (total 40)	37	40	40
SPPB (total 12)	9	11	12
6MWD	290 m	410 m	680 m
TGUG	12 sec	8.5 sec	8.2 sec

This case report confirms CARE case report guideline checklist (see Supplementary Checklist). Written informed consent was obtained from the patient for publication of the case details and accompanying images.

## 3 Discussion

Pulmonary symptoms including dyspnea, dry cough, dyspnea on exertion, and chest wall pain are reported manifestations of post-acute COVID-19 syndromes, so-called “long-COVID” or postacute sequelae of SARS-CoV-2 infection.^[1,7]^ It is generally agreed that COVID-19 complicates pulmonary fibrosis, whether temporary or permanent, as observed in SARS and middle east respiratory syndrome.^[2]^ Many pharmacological treatments have been challenged for postacute COVID-19 pulmonary fibrosis with limited evidence. Thus, rehabilitation is gaining more importance.

So far, no guidelines or systematic reviews for postacute COVID-19 pulmonary rehabilitation have been reported. Several narrative reviews suggested a pulmonary rehabilitation program based on the management guidelines of chronic obstructive pulmonary disease or other chronic pulmonary diseases.^[4,8]^ However, they did not provide the details of the rehabilitation program. In this case, we provided a standard and structured exercise program with intermittent monitoring. The consensus document on pulmonary rehabilitation in Korea is a standard pulmonary rehabilitation guideline for chronic obstructive pulmonary disease and pulmonary fibrosis patients in the Republic of Korea.

In general, pulmonary rehabilitation for lung fibrosis decreases dyspnea and improves exercise capacity and quality of life.^[9]^ However, it cannot reverse the anatomical changes and is not curative. In this case, after recovery from COVID-19 pneumonia, the patient did not complain of any neurological or musculoskeletal complications, except the need for continuous oxygen support for ambulation. Before undergoing rehabilitation, several attempts to wean the patient off oxygen support depressed him. Moreover, it was a great activity limitation and barrier for his discharge.

Based on the chest computed tomography (CT), after 10 rehabilitation sessions, the patient recovered rapidly, but the lung fibrosis was persistent; however, a reduction in the lung fibrosis was observed on the CT scan after 3 months of follow-up. This suggests that a 2-week rehabilitation program might decrease the length of hospital stay and help patients to get back into society.

During the pandemic, we experienced many similar cases. The shortage of medical supply and high infectivity of the virus made it impossible to provide necessary rehabilitation, thus contributing to a lack of evidence. We do not know the exact natural history nor aggravating or relieving factors of chronic fibrosis in COVID-19. Nonetheless, we insist on actively providing pulmonary rehabilitation programs for COVID-19 survivors. This case may be a shred of small evidence, but it will guide further prospective studies.

## Acknowledgments

The authors thank Jintae Jung, PT, Center for Rehabilitation Medicine, Seoul Medical Center, for his passionate provision of rehabilitation in this study. We also would like to thank Editage (www.editage.co.kr) for English language editing.

## Author contributions

**Conceptualization:** Kyungyeul Choi, JongKyu KIM.

**Data curation:** Kyungyeul Choi, Minwoo Kim, Son mi Lee.

**Investigation:** Minwoo Kim, Son mi Lee.

**Writing – original draft:** Kyungyeul Choi.

**Writing – review & editing:** JongKyu KIM.

## Correction

The orcid id appeared incorrectly when first published for Kyungyeul Choi and has been corrected to https://orcid.org/0000-0003-4218-0102.

## Supplementary Material

Supplemental Digital Content
