# Supplementary material for: Exercise-based pulmonary rehabilitation for a post-COVID-19 pulmonary fibrosis patient: A case report
Source: Medicine (Baltimore). 2021 Nov 24;100(47):e27980. doi: 10.1097/MD.0000000000027980 (PMC8615350; doi:10.1097/MD.0000000000027980)
Supplement: Supplemental Digital Content [file medi-100-e27980-s001.docx]

Supplementary 1. Pulmonary rehabilitation exercise protocol

| Frequency | 5 days a week |
| --- | --- |
| Intensity | Target intensity with 12 of Borg RPE  Maximal intensity with 14 of Borg RPE |
| Time & Type | 60 minutes a day |
|  | 5 minutes of stretching and warm-up |
|  | 5 minutes of strengthening exercises |
|  | including upper and lower extremities strengthening exercises* |
|  | 30 minutes of aerobic exercises |
|  | consist of treadmill walking and bicycle ergometer |
|  | 10 minutes of aerobic exercises |
|  | 10 minutes of cool down |
|  |  |
| Target SpO_2_** | Above 92% at resting |
|  | Above 87% during exercise |
|  |  |
| Goal | To complete the 6-minute walking test without oxygen supplementation while maintaining target SpO_2_ |

RPE, rating of perceived exertion

The exercise program was based on FITT (frequency, intensity, time, and time) principle.

*For upper extremity strengthening, dumbbells were used. For lower extremity strengthening, we used N-K (Noland and Kuckhoff) table with weights.

**SpO_2_ was detected via pulse oximeter attached on the index finger
